# Study on the characteristics of carotid wall shear stress in type 2 diabetes patients based on ultrasound vector flow imaging

**DOI:** 10.3389/fendo.2024.1409082

**Published:** 2024-11-21

**Authors:** Zhaohuan Li, Anguo Luo, Xuebing Liu, Shenghua Xie, Yulin Wang, Lan Deng, Shimin Zhong, Yaoxia Liu, Xu Cao, Yigang Du, Wen Luo, Yan Deng, Lixue Yin

**Affiliations:** ^1^ Department of Cardiovascular Ultrasound and Non-invasive Cardiology, Sichuan Provincial People’s Hospital, School of Medicine, University of Electronic Science and Technology of China, Chengdu, China; ^2^ Ultrasound in Cardiac Electrophysiology and Biomechanics Key Laboratory of Sichuan Province, Sichuan Clinical Research Center for Cardiovascular Disease, Sichuan Provincial People’s Hospital, School of Medicine, University of Electronic Science and Technology of China, Chengdu, China; ^3^ Department of Geriatric Endocrinology, Sichuan Provincial People’s Hospital, University of Electronic Science and Technology of China, Chengdu, China; ^4^ Department of Endocrinology, Sichuan Provincial People’s Hospital, University of Electronic Science and Technology of China, Chengdu, China; ^5^ Department of Ultrasound Research and Development, Shenzhen Mindray Bio-Medical Electronics Co., Ltd., Shenzhen, China; ^6^ Department of Clinical and Research, Shenzhen Mindray Bio-Medical Electronics Co., Ltd., Shenzhen, China

**Keywords:** carotid artery, wall shear stress, type 2 diabetes mellitus, vector flow imaging, cardiovascular diseases

## Abstract

**Objectives:**

We aimed to quantitatively analyze wall shear stress (WSS) of the common carotid artery (CCA) and elucidate the relationship between WSS and cardiovascular disease (CVD) in patients with type 2 diabetes mellitus (T2DM) using ultrasound vector flow (V-Flow) imaging.

**Methods:**

A total of 109 T2DM patients were selected as the DM group, while 49 healthy volunteers served as the control group. V-Flow examination of the bilateral CCA was conducted. The maximum wall shear stress (WSS_max_) and mean wall shear stress (WSS_mean_) at the bifurcation, proximal bifurcation and middle segment of the bilateral CCA were obtained.

**Results:**

The DM group showed decreased WSS_mean_ in the middle region and proximal bifurcation of the CCA compared with the control group (p < 0.05). The WSS_mean_ was further decreased in T2DM patients with CVD compared to those without CVD (middle region: 0.71 ± 0.17 Pa *vs.* 0.84 ± 0.24 Pa, p < 0.05; proximal bifurcation: 0.62 ± 0.22 Pa *vs.* 0.80 ± 0.21 Pa, p < 0.05). The receiver operating characteristic curve showed that a model combining with age, body mass index and WSS_mean_ at the proximal carotid bifurcation had diagnostic value for detecting CVD in T2DM patients (area under the curve: 0.862, p < 0.05).

**Conclusion:**

WSS_mean_ has potential value for evaluation of atherosclerosis, as well as in detecting the occurrence of CVD in T2DM patients. Ultrasound V-Flow imaging may be an effective tool for non-invasive evaluation of WSS in the clinic.

## Highlights

- The wall shear stress is useful for detecting and grading atherosclerosis, and have potential value for detecting CVD in T2DM.
- Ultrasound vector flow imaging (V-Flow) is an effective tool for non-invasive evaluation of wall shear stress in clinic.

## Introduction

Cardiovascular disease (CVD) is one of the most important factors affecting the prognosis of type 2 diabetes mellitus (T2DM) (1). The main pathological basis of diabetic cardiovascular disease is atherosclerosis, which can cause angina pectoris, myocardial infarction and stroke. Clinical observations and previous studies have shown that there is a disturbance in the blood flow field where the blood vessels curve and bifurcate, and these areas, which are usually associated with lower wall shear stress (WSS), are more prone to atherosclerosis (2–4). Therefore, accurate visualization and evaluation of the flow fields at these sites would be of great value in predicting the risk of atherosclerosis and assessing the degree of disease. WSS can be measured using magnetic resonance imaging (MRI), where an accurate three dimensional vascular shape can be obtained but the velocity estimation may have errors near the divider of the carotid bifurcation, leading to inaccurate WSS results (5). In addition, MRI is expensive, data processing is time-consuming, and MRI is not suitable for routine examination. Based on the conventional ultrasound Doppler technique, WSS has been calculated in several previous studies (6, 7). However, the errors were very large and only valid for laminar flow (7). It has been demonstrated that the errors are greatly reduced, particularly for complex flows compared to conventional pulsed wave Doppler, when WSS is calculated based on the vector velocities obtained using ultrasound vector flow (V-Flow) imaging (8). The vector velocities used in these WSS calculations have also been validated with different imaging techniques (MRI and computational fluid dynamics) in two comparison studies (9, 10). However, the WSS derived from V Flow imaging has not been verified extensively in the clinic, and current studies mainly focus on carotid stenosis (11). The purpose of this study was to observe the hydrodynamic state of the carotid artery in patients with T2DM by using the V-Flow technique and to explore the relationship between WSS and the occurrence of CVD.

## Methods

### Study population

**Figure 1:**
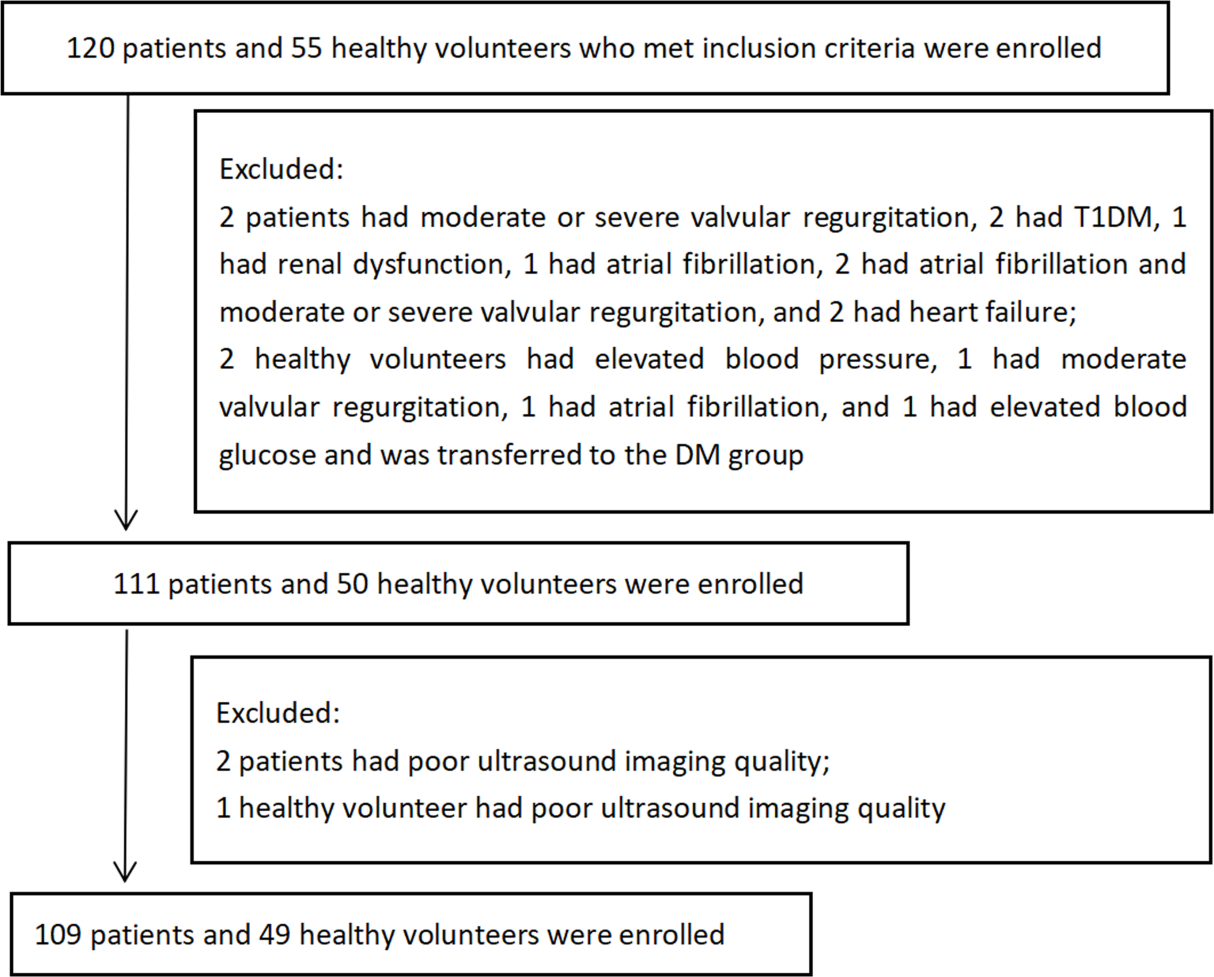
Flowchart describing the selection process for the study population.

A total of 109 patients with T2DM who were treated at Sichuan Provincial People’s Hospital from November 2018 to February 2019 were enrolled in this study as the DM group (**Figure 1**). The inclusion criteria of T2DM were those established by the American Diabetes Association (12). The exclusion criteria included type 1 diabetes mellitus, carotid artery stenosis ≥ 50%, cerebral hemorrhage, patent foramen ovale, phlebothrombosis, atrial fibrillation, valvular stenosis, valvular moderate or severe regurgitation, left ventricular ejection fraction (LVEF) < 50%, intracardiac thrombus, intracardiac tumor, carotid endarterectomy, familial hypercholesterolemia, Takayasu arteritis, systemic disease, liver or renal dysfunction and poor ultrasound imaging quality of the carotid artery (the poor quality of carotid ultrasound images was defined as the inability to identify the carotid intima of the observation segment, and the longitudinal view of the observation segment of the carotid artery did not completely pass through the central axis). The control group consisted of 49 healthy volunteers with no history of physical or laboratory evidence of DM, hypertension, hypercholesterolemia, or cardiovascular, cerebrovascular, or peripheral vascular diseases who were matched by age and sex to patients in the DM group. The exclusion criteria were the same as the DM group. This was a cross-sectional case-control study, in which the participants formed a convenience sample. The study protocol was approved by the ethics committees of Sichuan Provincial People’s Hospital, and informed consent was obtained from all participants.

### Demographic characteristics

Data on age, sex, body mass index (BMI), current or previous smoking, course of illness, administration of antidiabetic drugs and cardiovascular complications were recorded at enrollment. Serum levels of fasting plasma glucose (FPG), glycosylated hemoglobin A1c (HbA1c), serum triglycerides (TG), total cholesterol (TC), low-density lipoprotein cholesterol (LDL-c) and high-density lipoprotein cholesterol (HDL-c) were measured in the clinical laboratory department, and heart rate (HR) and upper limb artery blood pressure were measured on the day of the ultrasound examination.

### Carotid artery ultrasonography and image analysis

The examinations of bilateral carotid artery for all participants were conducted in a supine position. Mindray Resona 7 diagnostic ultrasound system (Shenzhen Mindray Bio-Medical Electronics Co., Shenzhen, China) and a L9-3U linear array probe (frequency 3.0-9.0 MHz) were used to scan the bilateral carotid arteries in the longitudinal-axis view, where the scanning range extended from the proximal end of the common carotid artery (CCA) to the carotid bifurcation.

Long-axis views of the middle segment of the CCA, which were defined according to the up- and down-poles of the thyroid, were scanned. The vascular cavity was kept horizontal and in the middle of the sampling box as much as possible. The patient was asked to keep still and to not swallow, and the V-Flow imaging mode was started. Then, the probe was held still for 1.5 s, while the software automatically processed the scan. Finally, the print button was clicked to store the V-Flow dynamic images. The V-Flow cine loops of the long-axis views at the bifurcation of the bilateral carotid arteries were collected by the same method.

**Figure 2:**
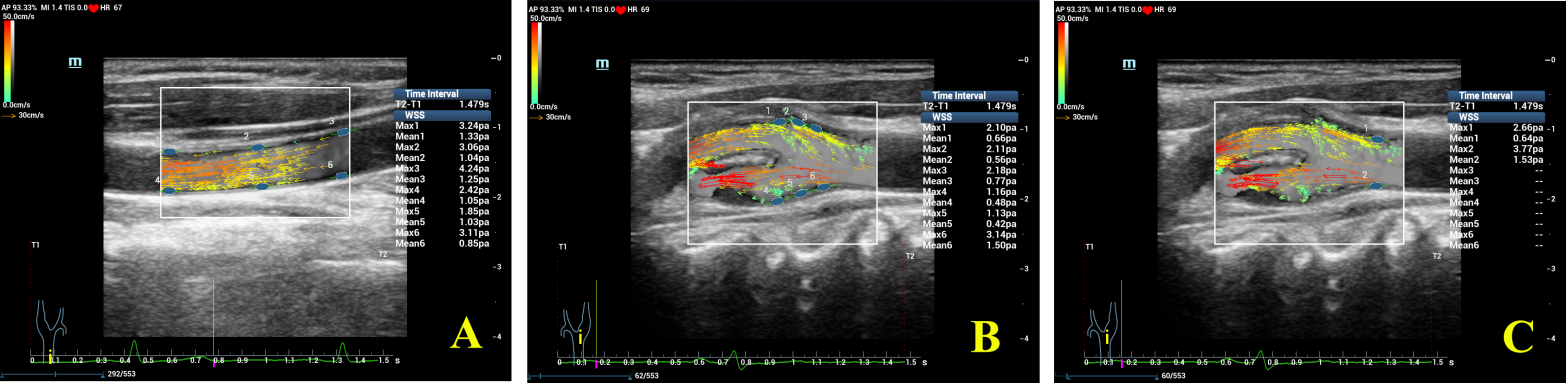
Detailed WSS measurements at different segments of the common carotid artery (CCA). **(A)** Measurement of WSS at the middle of the CCA; **(B)** measurement of WSS at the bifurcation of the CCA; **(C)** measurement of WSS at the proximal bifurcation of the CCA. Note that WSS at a maximum of 6 locations can be measured and shown in one image using V-Flow.

All the images were analyzed offline using the V-Flow function on the ultrasound system. Measurements of the carotid diameter (CD), peak velocity (V_p_), maximum and mean wall shear stress (WSS_max_ and WSS_mean_) were performed at the middle section, near the bifurcation and at the bifurcation of the CCA. WSS was measured at the middle of the CCAs by placing three sample points (overlapping the reference midline of the sample points over the intimal layer of the vessel wall and adjusting a correction line perpendicular to the vessel wall) separately at the front and back walls in the middle sections of the bilateral CCAs (**Figure 2A**) and then averaging the 12 measurements to obtain WSS_max_ and WSS_mean_ at the middle of the common carotid arteries for every subject. [It was previously demonstrated that there was no significant difference in WSS values between the right and left CCA (13)]. The measurement of WSS_max_ and WSS_mean_ at the bifurcation was identical to the measurement performed in the middle section (**Figure 2B**). WSS_max_ and WSS_mean_ were measured at the proximal bifurcation by placing one sample each at the front and back walls of the bilateral proximal carotid bifurcation (at the plaque-free far walls 1-2 cm proximal to the carotid bifurcation; **Figure 2C**), and then the four measurements were averaged to obtain WSS_max_ and WSS_mean_ at the proximal carotid bifurcation in every subject.

Plaque was defined as an intima-media thickness (IMT) ≥ 0.15 cm. The plaque burden was defined as the deviation of the extravascular membrane area and the endovascular membrane area divided by the extravascular membrane area.

At the long-axis views of the middle segment and the proximal bifurcation of the CCA, the sample box was placed in the area of interest, and the real-IMT mode was started. The mean value and standard deviation of IMT (IMT_mean_ and IMT_sd_) were calculated automatically.

### Subgrouping

The DM group was divided into DM + CVD and DM + non-CVD groups according to the presence or absence of CVD, respectively. Here, CVD included stable angina pectoris, unstable angina pectoris, myocardial infarction and stroke, which were verified according to the clinical diagnosis. The clinical diagnosis were blinded to the readers of the ultrasound images, while the results of the ultrasound image analysis were blinded to the assessors of the clinical diagnosis.

### Statistical analysis

Statistical analysis was performed with SPSS 21.0 (IBM, Armonk, NY, USA). Data are expressed as number (percentages) or mean ± SD. The Kolmogorov–Smirnov method was used to test the normality of the variables. The homogeneity of variance was assessed by the Levene method. Student’s *t*-test or Mann–Whitney test was used to compare two groups accordingly. Paired *t-*tests were used to compare the IMT_mean_ and IMT_sd_ in different segments of the CCAs. Analysis of variance or the Kruskal–Wallis test was used for multigroup comparisons, and Student–Newman–Keuls tests for all pairwise tests were used for intergroup comparisons. The chi-square test was applied to compare categorical data. Binary logistic regression was performed to select and validate potential diagnostic parameters for CVD in patients with T2DM. Both forward: LR selection (with probability for entry < 0.10) and backward: LR selection (with probability for entry and removal of 0.05 and 0.10, respectively) procedures were performed sequentially. In both procedures variables that have a significant impact on the regression model were retained in the combination models and the diagnostic value of ultrasound parameters for CVD was tested by receiver operating characteristic (ROC) analysis. A two-tailed p value < 0.05 was considered indicative of statistical significance.

## Results

### Demographic characteristics

There were no significant differences between the DM and control groups regarding age, sex or serum levels of TC and LDL-c. In patients with T2DM, current and previous smoking was more common; HR, systolic blood pressure (SBP), diastolic blood pressure (DBP), BMI and serum levels of TG, FPG and HbA1c were higher, while the serum level of HDL-c was lower than in the control group (**Table 1**). A total of 58/109 DM patients had 94 carotid plaques in all, and 93.62% of these plaques occurred at the bifurcation or proximal bifurcation. No plaques were found in the control group.

**Table 1:** Demographic and biochemical data for the DM and Control Groups.

	DM group (n=109)	Control group (n=49)	t/χ^2^	p
Age, y	63.33 ± 11.77	60.18 ± 10.54	1.604	0.111
Sex, male	55(50.5%)	27(55.1%)	0.292	0.589
BMI, kg/m^2^	25.01 ± 3.16	22.88 ± 2.89	3.842	**0.000**
SBP, mm Hg	142 ± 21	124 ± 17	5.375	**0.000**
DBP, mm Hg	79 ± 11	72 ± 7	4.438	**0.000**
HR, bpm	85 ± 13	79 ± 9	3.167	**0.002**
TG, mmol/L	2.05 ± 1.18	1.34 ± 0.56	4.550	**0.000**
TC, mmol/L	4.84 ± 1.19	4.49 ± 0.98	1.474	0.143
LDL-C, mmol/L	2.64 ± 0.91	2.51 ± 0.69	0.742	0.460
HDL-C, mmol/L	1.18 ± 0.35	1.33 ± 0.38	-2.127	**0.035**
FPG, mmol/L	10.36 ± 4.64	5.20 ± 0.48	11.401	**0.000**
HbA1c	9.68% ± 2.79%	5.20% ± 0.48%	15.273	**0.000**
Smoking, n (%)	36 (33.0%)	3 (6.1%)	13.162	**0.000**
Course of illness, y	8.84 ± 7.38	–	–	**-**
Administration of antidiabetic drugs				
Oral medicine	36 (33.0%)	–	–	**-**
Insulin	24 (22.0%)	–	–	**-**
Oral medicine+insulin	39(35.8%)	–	–	**-**
None	10 (9.2%)	–	–	**-**
Cardiovascular complications				
Stable angina pectoris	4 (3.7%)	–	–	**-**
Unstable angina pectoris	4 (3.7%)	–	–	**-**
Stroke	20 (18.3%)	–	–	**-**
Unstable angina pectoris and Stroke	4 (3.7%)	–	–	**-**

Data are expressed as means ± SD or as number (percentage).

BMI, body mass index; SBP, systolic blood pressure; DBP, diastolic blood pressure; HR, heart rate; TG, serum triglycerides; TC, total cholesterol; LDL-C, low-density lipoprotein cholesterol; HDL-C, high-density lipoprotein cholesterol; FPG, fasting plasma glucose; HbA1c, glycosylated hemoglobin A1c.

p < 0.05 highlighted in bold.

### Differences in ultrasound parameters among the different segments of the CCA

**Figure 3:**
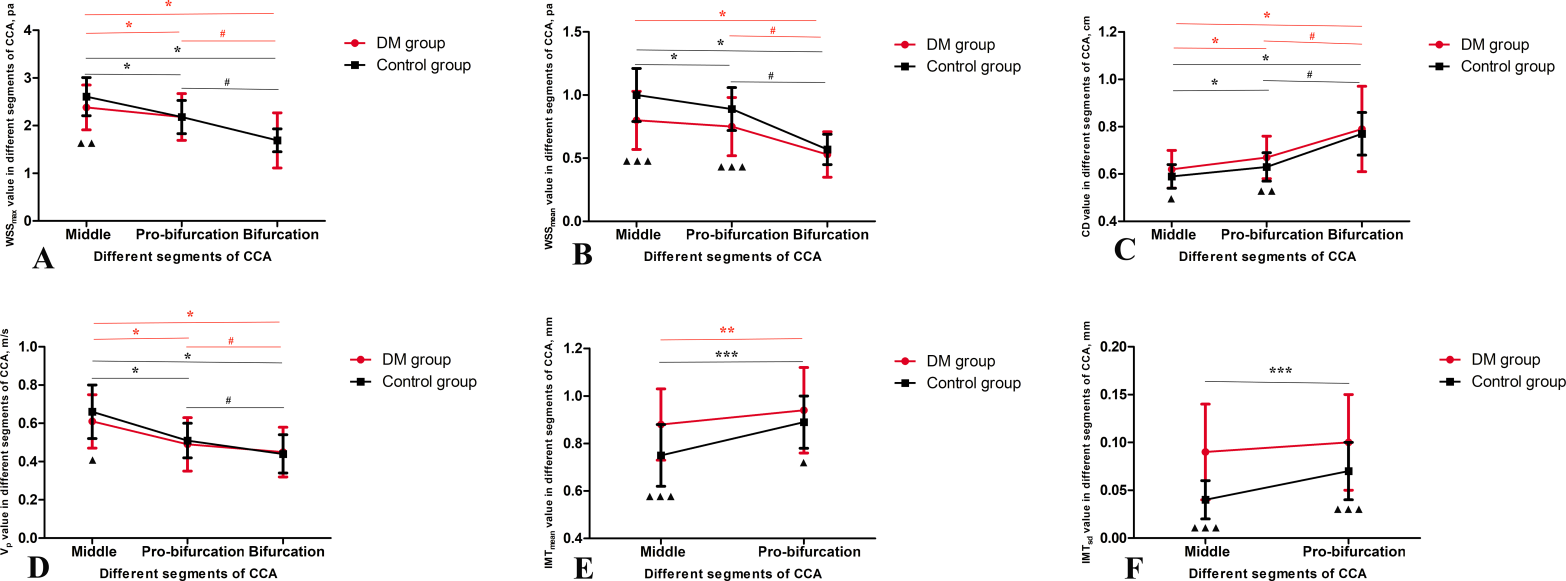
Ultrasound data in different segments of common carotid arteries (CCA) in the DM and control groups. **(A)** the maximum wall shear stress (WSS_max_), **(B)** the mean of the wall shear stress (WSS_mean_), **(C)** carotid diameter (CD), **(D)** peak velocity (V_p_), **(E)** the mean of the intima-media thickness (IMT_mean_) and **(F)** the standard deviation of the intima-media thickness (IMT_sd_). Data mean ± SD are presented. ▲p < 0.05, ▲▲p < 0.01, ▲▲p < 0.001 compared to control group; Red *p < 0.05 compared to the middle region in DM group; Red #p < 0.05 compared to the proximal bifurcation in DM group; Black *p < 0.05 compared to the middle region in control group; Black #p < 0.05 compared to the proximal bifurcation in control group.

WSS_max_ decreased gradually from the middle segment to the proximal bifurcation and then to the bifurcation of the CCA in both the DM group and the control group (all p < 0.05; **Figure 3A**, **Table 2**). In both groups, WSS_mean_ at the bifurcation was obviously lower than at the proximal bifurcation or at the middle of the CCA (all p < 0.05; **Figure 3B**, **Table 2**). In the control group, WSS_mean_ at the middle segment was obviously higher than at the proximal bifurcation of the CCA (p < 0.05; **Figure 3B**, **Table 2**). Although the WSS_mean_ in the middle segment was slightly higher than that at the proximal bifurcation in the DM groups, the difference was not significant (p > 0.05; **Figure 3B**, **Table 2**). In both groups, we also found that the CD was apparently enlarged at the bifurcation compared to the proximal bifurcation or the middle segment, and it was also enlarged at the proximal bifurcation compared to the middle segment (all p < 0.05; **Figure 3C**, **Table 2**). V_p_ was faster at the middle segment of the CCA in both groups than in the other two segments, while the V_p_ in the bifurcation was lowest among the three segments in both groups (all p < 0.05; **Figure 3D**, **Table 2**). The IMT_mean_ at the proximal bifurcation was thicker than that at the middle of the CCA in both groups (p < 0.05; **Figure 3E**, **Table 2**). The IMT_sd_ at the proximal bifurcation was increased compared to the middle region in the control group (p < 0.05), while there was no significant difference in the DM group (p > 0.05; **Figure 3F**, **Table 2**).

**Table 2:** Ultrasound data in different segments of common carotid arteries in the DM and control groups.

		DM group (n=109)	Control group (n=49)	t	p
WSS_max_, pa	The middle region	2.38 ± 0.47	2.61 ± 0.40	-3.049	**0.003**
	Proximal bifurcation	2.18 ± 0.49^*^	2.18 ± 0.35^*^	0.079	0.937
	The bifurcation	1.69 ± 0.58^*#^	1.69 ± 0.24^*#^	0.068	0.945
	F	51.026	92.050		
	p	**0.000**	**0.000**		
WSS_mean,_ pa	The middle region	0.80 ± 0.23	1.00 ± 0.21	-5.283	**0.000**
	Proximal bifurcation	0.75 ± 0.23	0.89 ± 0.17^*^	-4.274	**0.000**
	The bifurcation	0.53 ± 0.18^*#^	0.57 ± 0.12^*#^	-1.634	0.104
	F	50.291	81.376		
	p	**0.000**	**0.000**		
CD, cm	The middle region	0.62 ± 0.08	0.59 ± 0.05	2.432	**0.016**
	Proximal bifurcation	0.67 ± 0.09^*^	0.63 ± 0.06^*^	3.330	**0.001**
	The bifurcation	0.79 ± 0.18^*#^	0.77 ± 0.09^*#^	1.130	0.260
	F	53.549	95.543		
	p	**0.000**	**0.000**		
V_p_, m/s	The middle region	0.61 ± 0.14	0.66 ± 0.14	-2.083	**0.039**
	Proximal bifurcation	0.49 ± 0.14^*^	0.51 ± 0.09^*^	-1.374	0.171
	The bifurcation	0.45 ± 0.13^*#^	0.44 ± 0.10^*#^	0.110	0.913
	F	41.468	43.642		
	p	**0.000**	**0.000**		
IMT_mean_, mm	The middle region	0.88 ± 0.15	0.75 ± 0.13	5.343	**0.000**
	Proximal bifurcation	0.94 ± 0.18^**^	0.89 ± 0.11^***^	2.115	**0.036**
	t	-2.626	-5.815		
	p	**0.008**	**0.000**		
IMT_sd_, mm	The middle region	0.09 ± 0.05	0.04 ± 0.02	8.802	**0.000**
	Proximal bifurcation	0.10 ± 0.05	0.07 ± 0.03^***^	4.759	**0.000**
	t	-0.588	-5.410		
	p	**0.557**	**0.000**		

Data are expressed as means ± SD.

WSS_max_, maximum wall shear stress; WSS_mean_, mean of the wall shear stress; CD, carotid diameter; V_p_, peak velocity; IMT_mean_, mean of the intima-media thickness; IMT_sd_, standard deviation of the intima-media thickness.

^*^p < 0. 05, ^**^p < 0. 01, ^***^p < 0. 001 vs. the middle region; ^#^p < 0. 05 vs. the proximal bifurcation.

p < 0.05 highlighted in bold.

### Differences in ultrasound parameters between the DM and control groups

In the middle segment, the DM group showed lower WSS_max_ and WSS_mean_, wider CD, slower V_p_, thicker IMT_mean_ and increased IMT_sd_ than the control group (p < 0.05; **Figure 3**, **Table 2**). At the proximal bifurcation, the DM group displayed lower WSS_mean_, wider CD, thicker IMT_mean_ and increased IMT_sd_ compared to the control group (p < 0.05; **Figure 3**, **Table 2**). At the bifurcation, every ultrasound parameter was nearly the same in both groups (all p > 0.05; **Figure 3**, **Table 2**).

### Differences in demographic data and ultrasound parameters in three segments of the CCA among the DM + CVD Group, DM + non-CVD group and control group

**Figure 4:**
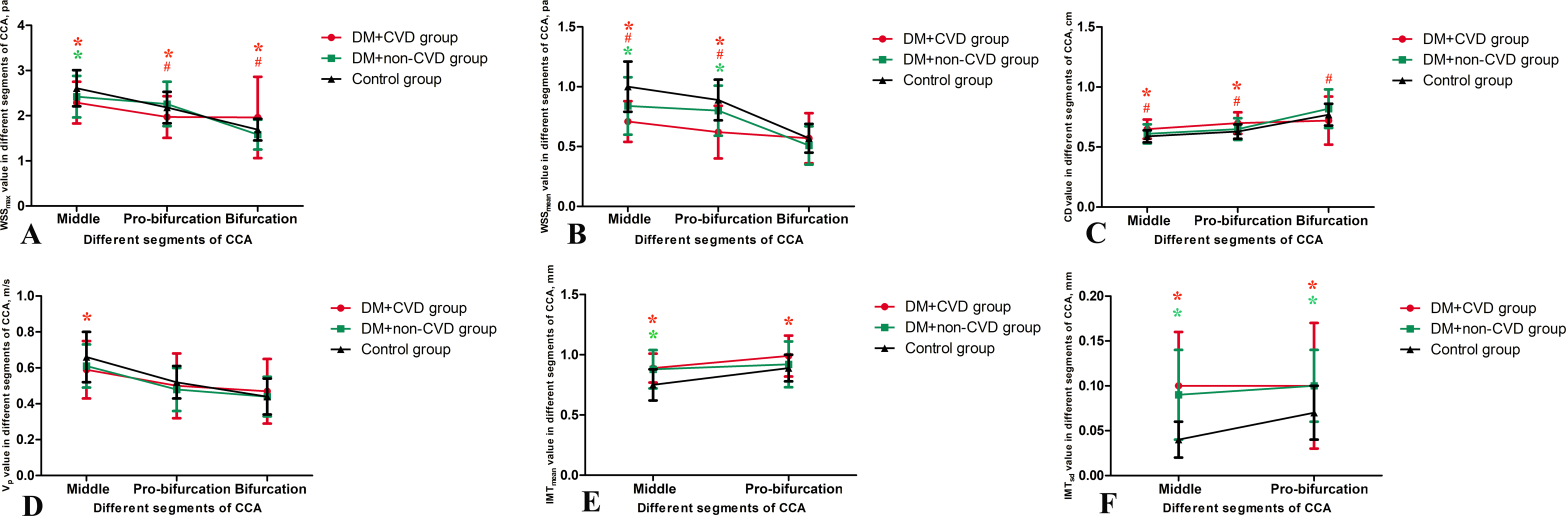
Ultrasound parameters in three segments of the common carotid artery (CCA) among the DM + CVD, DM + non-CVD and control groups. **(A)** the maximum wall shear stress (WSS_max_), **(B)** the mean of the wall shear stress (WSS_mean_), **(C)** carotid diameter (CD), **(D)** peak velocity (V_p_), **(E)** the mean of the intima-media thickness (IMT_mean_) and **(F)** the standard deviation of the intima-media thickness (IMT_sd_). Data mean ± SD are presented. Red *p < 0.05 the DM + CVD group compared to the control group; Red #p < 0.05 the DM + CVD group compared to the DM + non-CVD group; Green *p < 0.05 the DM + non-CVD group compared to the control group.

The demographic characteristics of the DM + CVD, DM + non-CVD and control groups are listed in **Table 3**. The DM + CVD group showed lower WSS_max_ and WSS_mean_, wider CD, slower V_p_, thicker IMT_mean_ and increased IMT_sd_ compared with the control group at the middle segment of the CCA, as well as lower WSS_max_ and WSS_mean_, wider CD, thicker IMT_mean_ and increased IMT_sd_ at the proximal bifurcation (p < 0.05; **Figure 4**, **Table 4**). Compared with the DM + non-CVD group, the DM + CVD group displayed lower WSS_max_ at the proximal bifurcation (p < 0.05; **Figure 4**, **Table 4**), and lower WSS_mean_ and wider CD both at the middle segment and proximal bifurcation (all p < 0.05; **Figure 4**, **Table 4**), but the two groups showed almost the same V_p_, IMT_mean_ and IMT_sd_ in each segment (p > 0.05; **Figure 4**, **Table 4**). In the DM + non-CVD group, the WSS_max_ at the middle segment was decreased, the WSS_mean_ at the middle segment and proximal bifurcation was lower, and the IMT_mean_ at the middle segment and IMT_sd_ at both of the middle segments and proximal bifurcation were larger than those in the control group (all p < 0.05; **Figure 4**, **Table 4**). The plaque burden was almost the same in the DM + CVD and DM + non-CVD groups (p > 0.05; **Table 4**).

**Table 3:** Demographic and biochemical data for the DM + CVD, DM + non-CVD and control groups.

	DM + CVD group (n=32)	DM + non-CVD group (n=77)	Control group (n=49)	F/χ^2^	p
Age, y	71.13 ± 6.79^*#^	60.09 ± 11.91	60.18 ± 10.54	13.637	**0.000**
Sex, male	16 (50.0%)	39 (50.6%)	27 (55.1%)	0.296	0.863
BMI, kg/m^2^	26.03 ± 3.19^*#^	24.57 ± 3.07^*^	22.88 ± 2.89	10.168	**0.000**
SBP, mm Hg	147 ± 20^*#^	138 ± 20^*^	122 ± 14	19.350	**0.000**
DBP, mm Hg	74 ± 11^#^	80 ± 10^*^	72 ± 7	11.240	**0.000**
HR, bpm	80 ± 12	82 ± 10	79 ± 9	1.189	0.307
TG, mmol/L	1.95 ± 0.86^*^	2.10 ± 1.31^*^	1.34 ± 0.57	5.629	**0.005**
TC, mmol/L	4.75 ± 1.46	4.88 ± 1.06	4.50 ± 0.98	1.210	0.302
LDL-C, mmol/L	2.49 ± 1.10	2.72 ± 0.80	2.52 ± 0.69	1.090	0.339
HDL-C, mmol/L	1.11 ± 0.26^*^	1.21 ± 0.38	1.33 ± 0.38	3.146	**0.046**
FPG, mmol/L	9.29 ± 3.87^*^	10.83 ± 4.90^*^	5.14 ± 0.47	30.552	**0.000**
HbA1c	9.03 ± 2.54^*^	9.98 ± 2.87^*^	5.20 ± 0.48	32.663	**0.000**
Smoking, n (%)	10 (31.3%)^**^	26 (33.8%)^***^	3 (6.1%)	13.239	**0.001**
Course of illness, y	9.49 ± 8.30	8.57 ± 7.01		0.575	0.566
Administration of antidiabetic drugs					
Oral medicine	10 (31.3%)	26 (33.8%)		0.615	0.893
Insulin	7 (21.9%)	17 (22.1%)			
Oral medicine+insulin	11 (34.4%)	28 (36.4%)			
None	4 (12.5%)	6 (7.8%)			
Cardiovascular complications					
Stable angina pectoris	4 (12.5%)	–	–	**-**	
Unstable angina pectoris	4 (12.5%)	–	–	**-**	
Stroke	20 (62.5%)	–	–	**-**	
Unstable angina pectoris and Stroke	4 (12.5%)				

Data are expressed as means ± SD or as number (percentage).

BMI, body mass index; SBP, systolic blood pressure; DBP, diastolic blood pressure; HR, heart rate; TG, serum triglycerides; TC, total cholesterol; LDL-C, low-density lipoprotein cholesterol; HDL-C, high-density lipoprotein cholesterol; FPG, fasting plasma glucose; HbA1c, glycosylated hemoglobin A1c.

^*^p < 0. 05, **p < 0. 01, ***p < 0. 001 vs. the control group; #p < 0. 05, vs. the DM + non-CVD group.

p < 0.05 highlighted in bold.

**Table 4:** The differences in ultrasound parameters in three segments of the common carotid artery for the DM + CVD, DM + non-CVD and control groups.

		DM +CVD group (n=32)	DM + non-CVD group (n=77)	Control group (n=49)	F/t	p
WSS_max_, pa	The middle region	2.29 ± 0.46^*^	2.42 ± 0.46^*^	2.61 ± 0.40	5.646	**0.004**
	Proximal bifurcation	1.97 ± 0.46^*#^	2.26 ± 0.49	2.18 ± 0.35	4.906	**0.009**
	The bifurcation	1.96 ± 0.90^*#^	1.58 ± 0.33	1.69 ± 0.24	6.828	**0.001**
WSS_mean_, pa	The middle region	0.71 ± 0.17^*#^	0.84 ± 0.24^*^	1.00 ± 0.21	18.629	**0.000**
	Proximal bifurcation	0.62 ± 0.22^*#^	0.80 ± 0.21^*^	0.89 ± 0.17	17.318	**0.000**
	The bifurcation	0.57 ± 0.21	0.51 ± 0.16	0.57 ± 0.12	2.750	0.067
CD, cm	The middle region	0.65 ± 0.08^*#^	0.61 ± 0.08	0.59 ± 0.05	7.336	**0.001**
	Proximal bifurcation	0.70 ± 0.09^*#^	0.65 ± 0.09	0.63 ± 0.06	8.235	**0.000**
	The bifurcation	0.72 ± 0.20^#^	0.82 ± 0.16	0.77 ± 0.09	4.817	**0.009**
V_p_, m/s	The middle region	0.59 ± 0.16^*^	0.61 ± 0.12	0.66 ± 0.14	2.604	0.077
	Proximal bifurcation	0.50 ± 0.18	0.48 ± 0.12	0.52 ± 0.09	1.101	0.335
	The bifurcation	0.47 ± 0.18	0.44 ± 0.11	0.44 ± 0.10	0.759	0.470
IMT_mean_, mm	The middle region	0.89 ± 0.12^*^	0.88 ± 0.16^*^	0.75 ± 0.13	14.201	**0.000**
	Proximal bifurcation	0.99 ± 0.17^*^	0.92 ± 0.19	0.89 ± 0.11	3.357	**0.037**
IMT_sd_, mm	The middle region	0.10 ± 0.06^*^	0.09 ± 0.05^*^	0.04 ± 0.02	23.562	**0.000**
	Proximal bifurcation	0.10 ± 0.07^*^	0.10 ± 0.04^*^	0.07 ± 0.03	7.693	**0.001**
Plaque burden, %		23.32 ± 6.56	22.92 ± 4.12	_	0.268	0.789

Data are expressed as means ± SD.

WSS_max_, maximum wall shear stress; WSS_mean_, mean of the wall shear stress; CD, carotid diameter; V_p_, peak velocity; IMT_mean_, mean of the intima-media thickness; IMT_sd_, standard deviation of the intima-media thickness.

^*^Compared to the control group, p < 0.05; ^#^Compared to the DM + non-CVD group, p < 0.05.

p < 0.05 highlighted in bold.

### Potential diagnostic parameters for CVD in T2DM

The demographic data and ultrasound parameters that were different between the DM + CVD group and the DM + non-CVD group, were processed via a binary logistic regression model. After validation by both forward and backward LR selection, only age, BMI and WSSmean at the proximal bifurcation remained significant in the regression model (**Table 5**).

**Table 5:** Binary logistic regression in DM + CVD and DM + non-CVD groups.

Factors	*β*	SE	Wals	p	OR (95% CI)
Age	0.138	0.037	13.991	0.000	1.148 (1.068-1.235)
BMI	0.274	0.094	8.536	0.003	1.315 (1.094-1.580)
WSS_mean_ at the proximal bifurcation	-3.384	1.453	5.427	0.020	0.034 (0.002-0.584)
Constant	-14.516	3.995	13.205	0.000	

**Figure 5:**
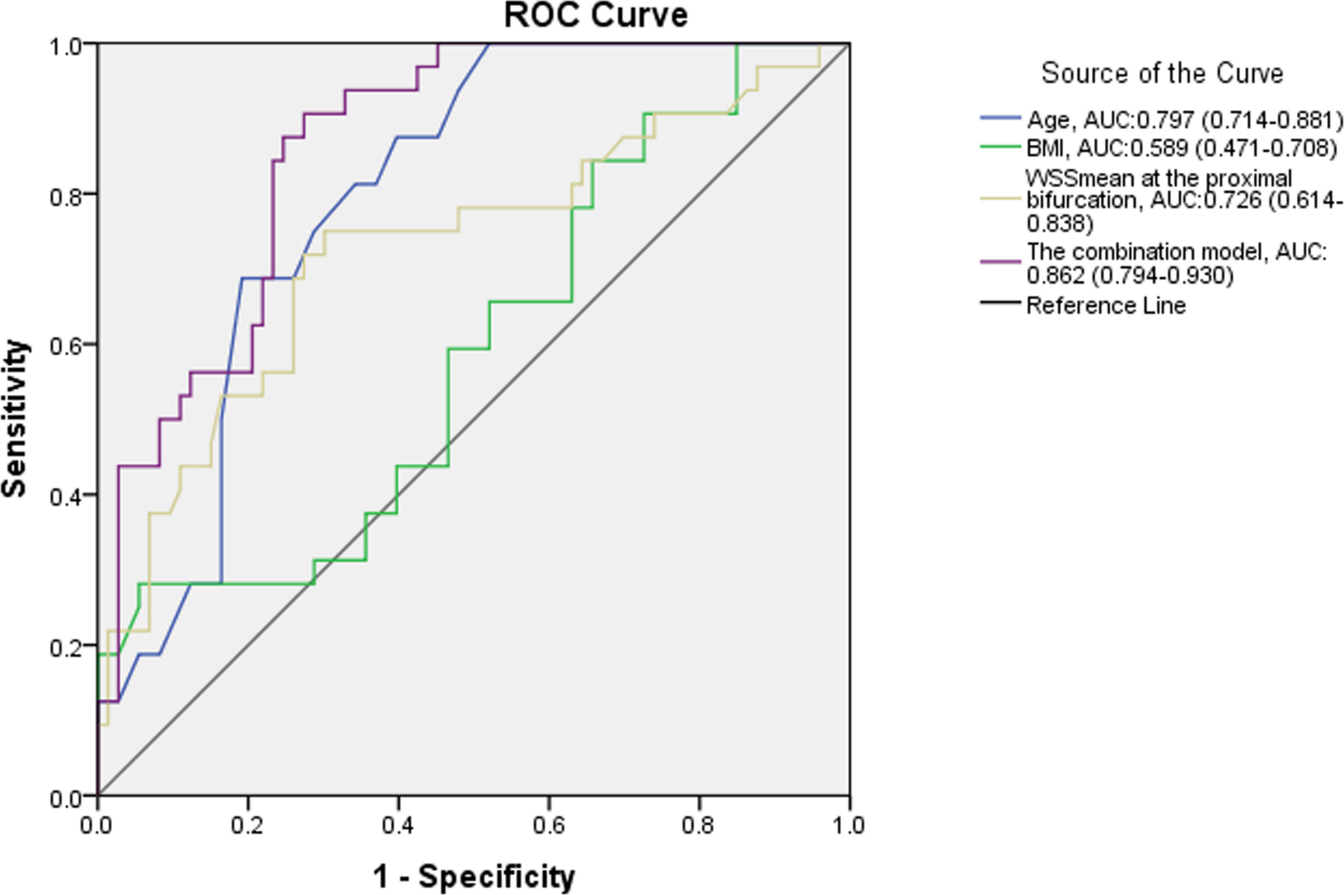
Receiver operating characteristic analysis of WSS_mean_ for differentiating CVD in T2DM patients.


Based on the above results, diagnostic models combining age, BMI and WSS_mean_ at the proximal bifurcation for CVD were developed. The areas under the receiver operating characteristic (AUC) curves for age, BMI and WSS_mean_ at the proximal bifurcation and combination model (combined the age, BMI and WSSmean at the proximal bifurcation) were 0.797, 0.589, 0.726 and 0.862, respectively (**Figure 5**). Comparing the diagnostic values of any two single parameters, only age and BMI had obviously different performance for the diagnosis of CVD, as shown by their AUC curves (p = 0.009). The combination model compared to any single parameter yielded the largest AUC of 0.862 (all p < 0.05), with a probability formula for differentiating with CVD from those without CVD in the DM group as follows:

$$P=\frac{e^{-14.516+0.138X1+0.274X2-3.384X3}}{1-e^{-14.516+0.138X1+0.274X2-3.384X3}}$$

where X1, X2 and X3 are age, BMI and WSS_mean_ at the proximal bifurcation, respectively. The sensitivity and specificity of this combination model were 90.6% and 72.6%, respectively, when 0.206 was taken as the diagnostic cutoff value of predictive probability.

## Discussion

In this study, we found that the WSS_mean_ in T2DM patients was decreased at the middle segment and at the proximal bifurcation of the CCA compared to the controls, and it was further decreased in T2DM patients with CVD compared to those without CVD; furthermore, the model combining age, BMI and WSS_mean_ at the proximal carotid bifurcation had diagnostic value for detecting CVD in T2DM. This study showed that V-Flow imaging may be a potentially valuable technology to detect WSS in the clinic.

WSS expresses the frictional force per unit area exerted by a fluid (i.e., the blood flow) in a direction parallel to the flow on the local tangent plane of the vessel wall (14). The WSS is calculated according to the general equation $\tau =\mu \cdot {\text{d}u/\text{d}y|}_{\text{wall}}$ , where μ is blood viscosity, u is flow velocity, y is the distance from the surface and du/dy is the velocity gradient, also called the shear rate (8). The WSS is the multiplication of the wall shear rate and the blood viscosity. WSS, a mechanical signal, can be perceived by vascular endothelial cells and transmitted within the cell, triggering activity in a series of cell signaling pathways (15, 16). It is well known that low WSS promotes atherosclerosis, which may be because low WSS can cause the high expression of multiple adhesion molecules that lead to an increase in lipid transendothelial transport and subendothelial deposition, trigger inflammatory pathways such as leukocyte adhesion and foam cell formation and eventually cause plaque development through the atherosclerosis cascade (17, 18). Therefore, detecting arterial WSS in clinical practice may be helpful in the prediction and evaluation of atherosclerosis.

V-Flow imaging, which is implemented based on steered plane wave and interleaved focus wave transmissions (19, 20), can estimate the 2D vector velocity by reconstructing multiple velocity components at a higher frame rate. This makes it possible to provide a better spatiotemporal vector of blood flow, especially in the regions close to the vessel wall, where both the velocity magnitude and direction can be measured with angle independence, for an optimal measurement of WSS that does not involve the use of invasive techniques or computer simulation (21). Our previous study showed that V-Flow imaging has good interobserver and intraobserver agreement [ICCs: 0.75–0.92, p < 0.05 (22)], that was also validated in two previous studies with the ICC (13) and Kendall W coefficient (11), respectively.

### The characteristics of WSS distribution in the carotid artery

In this study, WSS decreased gradually from the middle region of the CCA to the bifurcation in both groups, which was accompanied by enlarged CD and reduced Vp. However, Qiu et al. found no differences in WSS in different segments of the CCA (13). This inconsistency may have resulted from the different populations of subjects used in the studies. In the present study, most subjects were over 60 years old, while in Qiu’s study, only 15% were over 60 years old. Reduced WSS is usually associated with longer retention of blood flow, which can increase the contact time of platelets, macrophages and other atherosclerosis-causing factors, resulting in endothelial dysfunction and increased lipoprotein deposition in the vascular wall, causing abnormal vascular dilation and atherosclerosis (23). These factors might explain why 93.62% of plaques occurred near the bifurcation in this study. In the control group, WSS at the carotid bifurcation was also decreased compared to other segments of the CCA, but no plaques developed. To explain this phenomenon, more attention should be paid to the difference in amplitude between the control group and the DM group.

### Characteristics of WSS of the carotid artery in T2DM patients

A wider carotid diameter means that vascular remodeling has occurred in T2DM patients and is associated with slower flow velocity. T2DM patients appear to have a thicker and rougher carotid intima-media, which could be a sign of atherosclerosis. Obviously decreased WSS may be one of the factors that increase the risk of atherosclerosis in T2DM patients. However, these changes seem to be focused only on the middle segment and proximal bifurcation of the CCA. None of these ultrasound parameters showed significant differences at the carotid bifurcation. We consider that this may be associated with plaque location. The plaques were mainly located at the carotid bifurcation, which could shorten the regional diameter and increase flow velocity and eventually increase WSS. It thus may be a compensatory response of the organism to low WSS.

### Relationship of WSS and CVD in T2DM patients

In subgroup analysis, CD was widened in T2DM patients with CVD, but was not changed in T2DM patients without CVD compared to the control group. This finding suggests that CD may not be responsive to atherosclerosis in T2DM. IMT_mean_ and IMT_st_ were increased in T2DM, regardless of the presence or absence of CVD, compared to the control group, but they were not significantly different between the two T2DM groups. IMT_mean_ and IMT_st_ may reflect atherosclerosis in T2DM, but it would be difficult to grade atherosclerosis from these measures. V_p_ was almost the same among the three groups. WSS_max_ and WSS_mean_ may thus be more useful parameters for both detecting and grading atherosclerosis because they are decreased in T2DM patients without CVD and are further decreased in those with CVD in some segments of the CCA. The ROC curve revealed that among all different demographic data and ultrasound parameters between the groups of DM with and without CVD in this study, only age, BMI and WSS_mean_ at the proximal bifurcation had potential diagnostic value for detecting the occurrence of CVD in T2DM patients. The combination diagnostic value was apparently increased over any single parameter. Therefore, WSS_mean_ may be a more valuable parameter for the clinical evaluation of atherosclerosis than IMT, CD, V_p_ and WSS_max_.

At the proximal bifurcation, the blood flow changes from stable laminar flow to a non-laminar flow state, which causes turbulent flow and is usually associated with a lower WSS_mean_. For the healthy person, the WSS at the proximal bifurcation is also decreased compared to the middle segment of the CCA, but plaques did not develop. As the WSS decreased significantly in T2DM patients, the characteristics of atherosclerosis including plaque formation appeared, and the more decreased WSS was associated with a greater probability of CVD. This again indicated that low WSS is significantly correlated with an accelerated atherosclerosis progression (24, 25). The low WSS of T2DM patients may aggravate damage to carotid endothelial cells, promote the carotid IMT, and result in the formation of carotid atherosclerotic plaques which further contribute to the increased risk of cardiovascular and cerebrovascular diseases (25, 26). Moreover, a previous study has indicated that carotid WSS could be used as an indicator of systemic atherosclerosis and predict coronary atherosclerosis (27).

WSS_mean_ may be a more valuable parameter for evaluating atherosclerosis than CD, V_p_, IMT_mean_, IMT_sd_ and WSS_max_, which has potential clinical value for cardiovascular risk stratification and classification therapy for patients with T2DM.

### Study limitations

A cross-sectional study cannot accurately explore the predicted value of WSS for CVD, only the association between them. A small sample size limits the ability to perform deep subgroup analysis. A prospective study with a large sample size is needed to assess the value of WSS in cardiovascular risk prediction in patients with T2DM. We did not evaluate WSS around the plaques and its relationship to CVD, which is a complicated issue, and we intend to address in another study.

## Conclusions

WSS at the plaque-free wall of the CCA may be a useful parameter for both detecting and grading atherosclerosis in T2DM patients. A model combining age, BMI and WSS_mean_ at the proximal carotid bifurcation may have diagnostic value for detecting CVD in T2DM. Ultrasound V-Flow imaging may be an effective tool for non-invasive evaluation of atherosclerosis in the clinic.

## Data Availability

The original contributions presented in the study are included in the article/**Supplementary Material**. Further inquiries can be directed to the corresponding authors.
