# Supplementary material for: Study on the characteristics of carotid wall shear stress in type 2 diabetes patients based on ultrasound vector flow imaging
Source: Front Endocrinol (Lausanne). 2024 Nov 21;15:1409082. doi: 10.3389/fendo.2024.1409082 (PMC11617173; doi:10.3389/fendo.2024.1409082)
Supplement: Supplementary file 1 [file Presentation1.pdf]

Supplementary material 1: Subgroup analysis was performed to eliminate the interference of comorbidity on the analysis of carotid fluid dynamics parameters of type 2 diabetes mellitus.

The subgroup analysis is detailed: divided the DM group into DM with comorbidity group and DM without comorbidity group. The results showed that the only difference in demographic and biochemical data between DM without comorbidity group and control group was blood glucose index (HbA1c and FPG) (Table 1). Moreover, there were still differences in WSS_{mean} in the middle segment and near the bifurcation, and WSSmax in the middle segment between DM without comorbidity group and control group (Table 2), indicating that patients with only elevated blood glucose had significant lower carotid artery WSS than healthy people.

Table 1 Demographic and Biochemical Data among the DM with Comorbidity Group, DM without Comorbidity Group and Control Groups

	DM with comorbidity group (n=79)	DM without comorbidity group (n=30)	Control group (n=49)
Age, y	63.18±11.48	63.73±12.71	60.18±10.54
Sex, male	43 (54.4%)	12 (40%)	27 (55.1%)
BMI, kg/m2	25.81±2.86$^{*\#}$	22.58±2.84	22.88±2.89
SBP, mm Hg	145±20$^{*\#}$	128±15	122±14
DBP, mm Hg	79±11$^{*\#}$	74±7	72±7
HR, bpm	81±10	83±12	79±9
TG, mmol/L	2.22±1.23$^{*\#}$	1.47±0.76	1.34±0.57
TC, mmol/L	4.76±1.24	5.11±0.99	4.50±0.98
LDL-C, mmol/L	2.56±0.94	2.91±0.76	2.52±0.69

HDL-C, mmol/L	1.12±0.34*	1.39±0.30	1.33±0.38
FPG, mmol/L	10.19±4.98*	10.91±3.47*	5.14±0.47
HbA1c	9.68±2.91*	9.71±2.45*	5.20±0.48
Smoking, n (%)	33 (41.8%)***##	3 (10.0%)	3 (6.1%)

Data are expressed as the mean ± SD or as number (percentage).

BMI: body mass index; SBP: systolic blood pressure; DBP: diastolic blood pressure; HR: heart rate; TG: serum triglycerides; TC: total cholesterol; LDL-C: low-density lipoprotein cholesterol; HDL-C: high-density lipoprotein cholesterol; FPG: fasting plasma glucose; HbA1c, glycosylated hemoglobin A1c.

* Compared to the control group, $P < 0.05$, # Compared to the DM without comorbidity group, $P < 0.05$

Table 2 The Differences in Ultrasound Parameters in Three Segments of the Common Carotid Artery among the DM with Comorbidity, DM without Comorbidity and Control Groups

		DM with comorbidity group (n=79)	DM without comorbidity group (n=30)	Control group (n=49)
	The middle segment	2.39±0.46*	2.34±0.50*	2.61±0.40
WSS_{max}, pa	Near the bifurcation	2.18±0.52	2.17±0.42	2.18±0.35
	The bifurcation	1.73±0.65	1.59±0.30	1.69±0.24
	The middle segment	0.78±0.20*	0.85±0.28*	1.00±0.21
WSS_{mean}, pa	Near the bifurcation	0.74±0.26*	0.75±0.20*	0.89±0.17
	The bifurcation	0.52±0.18	0.53±0.17	0.57±0.12
CD, cm	The middle segment	0.64±0.08*	0.58±0.09	0.59±0.05

	Near the bifurcation	0.67±0.10*	0.65±0.06	0.63±0.06
	The bifurcation	0.79±0.18	0.79±0.18	0.77±0.09
	The middle segment	0.62±0.12	0.58±0.17*	0.66±0.14
Vp, cm/s	Near the bifurcation	0.49±0.15	0.48±0.11	0.52±0.09
	The bifurcation	0.45±0.14	0.43±0.10	0.44±0.10
IMT_{max}, mm	The middle segment	0.98±0.19*	0.96±0.17*	0.84±0.13
	Near the bifurcation	1.14±0.20*	1.10±0.18*	1.01±0.16
IMT_{mean}, mm	The middle segment	0.90±0.14*	0.84±0.17*	0.75±0.13
	Near the bifurcation	0.94±0.19	0.95±0.18	0.89±0.11
IMT_{sd}, mm	The middle segment	0.09±0.05*	0.11±0.05*	0.04±0.02
	Near the bifurcation	0.10±0.05*	0.09±0.04*	0.07±0.03

Data are expressed as the mean ± SD.

WSSmax: the maximum wall shear stress; WSSmean: the mean of the wall shear stress; CD: carotid diameter; Vp: peak velocity; IMTmean: the mean of the intima-media thickness; IMTsd: the standard deviation of the intima-media thickness.

* Compared to the control group, P < 0.05, # Compared to the DM without comorbidity group, P < 0.05

Supplementary material 2: To investigate the changes of IMT_{max} among the groups

We collated and analyzed the IMT_{max} data and discovered that its trend was consistent with IMT_{mean} among different groups (Table 3 and Table 4).

Table 3. IMT_{max} in Different Segments of Common Carotid Arteries in the DM and Control Groups.

	DM group (n=109)	Control group (n=49)	t	p

IMT$_{max}$, mm	The middle region	0.97 ± 0.19	0.84 ± 0.13	5.258	**0.000**
	Proximal bifurcation	1.13 ± 0.20	1.01 ± 0.16	3.914	**0.000**
	t	-7.173	-8.122		
	p	**0.000**	**0.000**		

Data are expressed as the mean ± SD.

IMT$_{max}$: maximum of the intima-media thickness.

Table 4. IMT$_{max}$ in Different Segments of Common Carotid Artery in the DM + CVD, DM + non-CVD and Control Groups.

		DM +CVD group (n=32)	DM + non-CVD group (n=77)	Control group (n=49)	F/t	p
IMT$_{max}$, mm	The middle region	0.99 ± 0.19*	0.97 ± 0.19*	0.84 ± 0.13	10.698	**0.000**
	Proximal bifurcation	1.17 ± 0.21*	1.11 ± 0.19*	1.01 ± 0.16	7.659	**0.001**

Data are expressed as the mean ± SD.

IMT$_{mean}$: mean of the intima-media thickness.

* Compared to the control group, $p < 0.05$; # Compared to the DM + non-CVD group, $p < 0.05$.
